# Degree of islet function preservation and continuous glucose monitoring in individuals undergoing total pancreatectomy with islet autotransplantation

**DOI:** 10.1007/s00125-026-06772-9

**Published:** 2026-06-27

**Authors:** Maarten C. Tol, Willemijn E. M. E. de Vos, Ian P. J. Alwayn, J. Sven D. Mieog, Jeanin E. van Hooft, Akin Inderson, Marieke Niesters, Marieke D. Hellinga, Michiel F. Nijhoff, Arian R. van Erkel, Marten A. Engelse, Volkert A. L. Huurman, Eelco J. P. de Koning

**Affiliations:** 1 Department of Internal Medicine, Leiden University Medical Center, Leiden, the Netherlands; 2 LUMC Transplant Center, Leiden University Medical Center, Leiden, the Netherlands; 3 Department of Surgery, Leiden University Medical Center, Leiden, the Netherlands; 4 Department of Gastroenterology and Hepatology, Leiden University Medical Center, Leiden, the Netherlands; 5 Department of Anaesthesiology, Leiden University Medical Center, Leiden, the Netherlands; 6 Department of Radiology, Leiden University Medical Center, Leiden, the Netherlands

**Keywords:** Autologous islet transplantation, Continuous glucose monitoring, Glycaemic control, Islet function, Mixed meal tolerance test, Total pancreatectomy, TPIAT

## Abstract

**Aims/hypothesis:**

In individuals undergoing total pancreatectomy, an additional islet autotransplantation (total pancreatectomy with islet autotransplantation [TPIAT]) can be performed. We investigated the degree to which islet secretory function is preserved after TPIAT and assessed the relation to glycaemic control using continuous glucose monitoring.

**Methods:**

Eligibility for TPIAT was assessed by a multidisciplinary team. The cohort consisted of all individuals undergoing TPIAT from 2014 to 2024. To assess islet secretory function, participants were subjected to a 2 h liquid meal stimulation test prior to TPIAT, at 3 months and annually post TPIAT. Glycaemic control was assessed using continuous glucose monitoring and HbA_1c_.

**Results:**

Twenty-six individuals were included, of whom 88.5% had chronic pancreatitis. At baseline, 57.7% had prediabetes (HbA_1c_ 39–47 mmol/mol [5.7–6.4%] or a fasting glucose 5.6–6.9 mmol/l) or diabetes, and 46.2% had undergone previous pancreatic surgery. Islet secretory function determined by C-peptide area under the curve (AUC_C-peptide_) was 45% of baseline at 3 months post TPIAT. Preservation of islet secretory function was independent of preoperative glycaemic status and previous surgery. At 3 months, time in range was 75.2 ± 26.1%, and time in range was positively correlated with AUC_C-peptide_ (*p*<0.0001). After 3 months islet function did not significantly change, which was reflected in a stable BETA-2 score.

**Conclusions/interpretation:**

Nearly half of islet secretory function is retained after TPIAT. These results can facilitate shared decisions on the potential benefits of islet autotransplantation after total pancreatectomy, even in individuals with (pre)diabetes and previous pancreatic surgery.

**Graphical Abstract:**

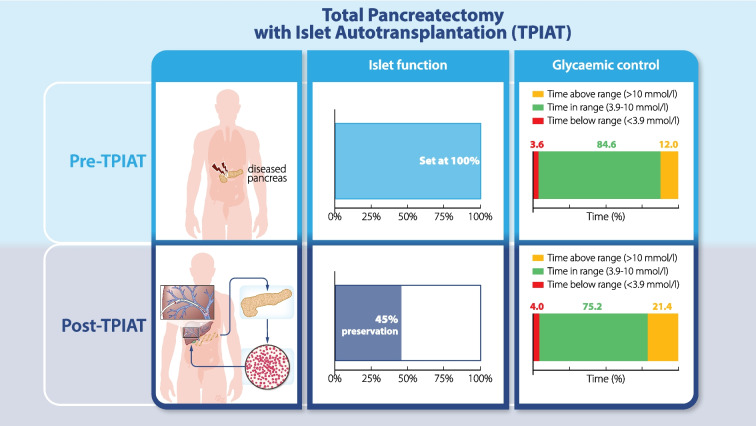

**Supplementary Information:**

The online version contains peer-reviewed but unedited supplementary material available at 10.1007/s00125-026-06772-9.

## Introduction


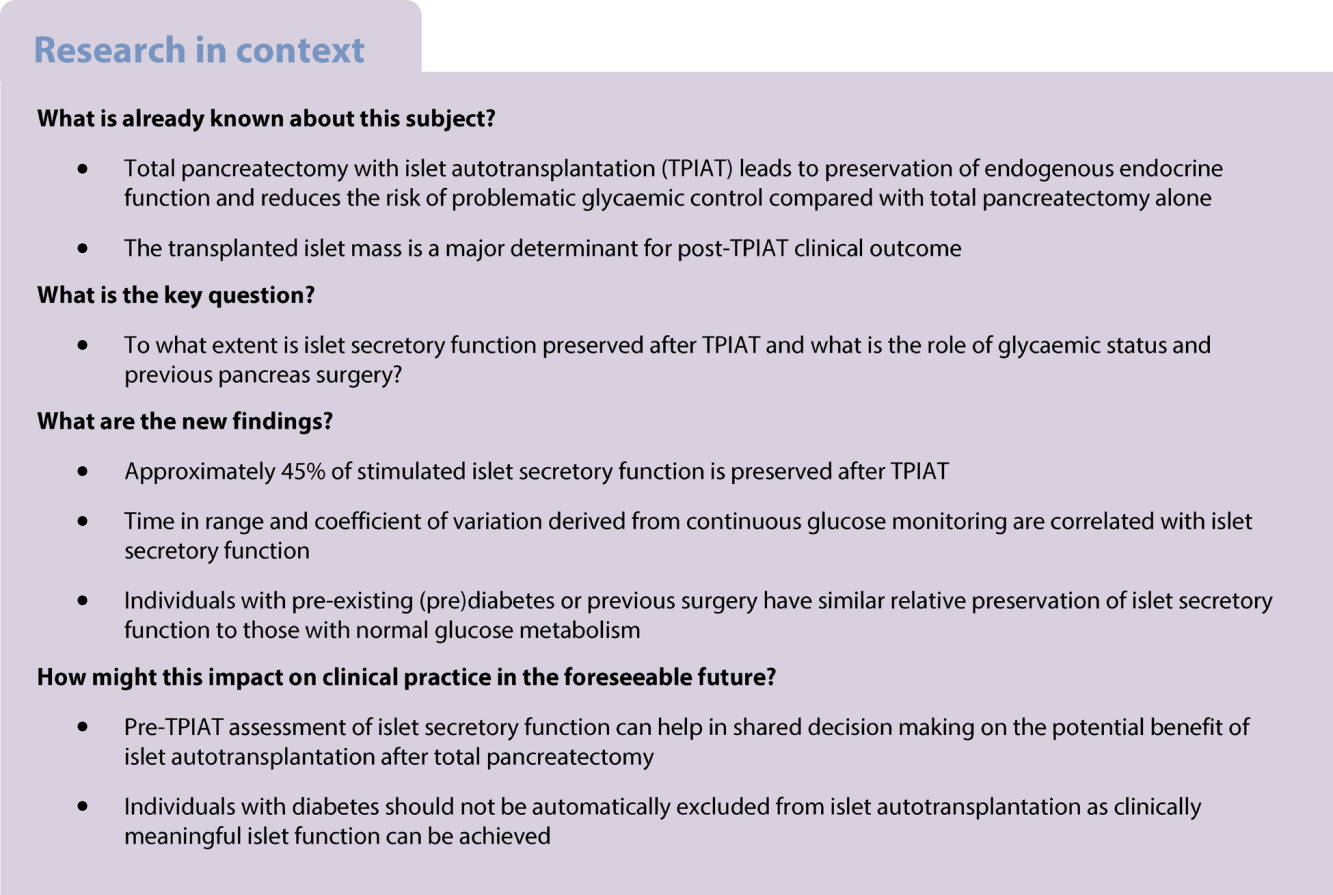


Total pancreatectomy with islet autotransplantation (TPIAT) is primarily indicated for non-malignant pancreatic diseases such as chronic pancreatitis [1], in which repetitive episodes of pancreatic inflammation lead to irreversible damage of the pancreas and chronic pain [2]. Consequently, individuals will have reduced islet secretory function, manifesting as prediabetes or diabetes mellitus 3c. Risk factor management, such as cessation of alcohol consumption and smoking, together with symptomatic treatment such as pancreatic duct dilation and optimisation of analgesics, precede several surgical treatments, with total pancreatectomy being a last resort option. The goal of TPIAT is to improve quality of life and preserve endogenous functional endocrine mass through autologous transplantation of the pancreatic islets, thereby achieving more stable glycaemic control and lower risk of diabetes-related complications compared with total pancreatectomy alone [3–5]. Preserved islet secretory function is associated with a reduced risk of microvascular complications and (severe) hypoglycaemic events [6–10]. In addition, islet autotransplantation can lead to a simplified insulin regimen (such as only long-acting insulin) or no insulin at all, improving the treatment burden of diabetes.

Typical endpoints to assess treatment success in studies on TPIAT are HbA_1c_ and insulin requirement [4, 11–14]. Insulin independence is the optimal outcome, reached by 20–30% of individuals after autologous islet transplantation [4, 15, 16]. Glycaemic control is influenced by islet secretory function in combination with several other factors, such as peripheral insulin sensitivity, medication use and lifestyle. In clinical practice, islet secretory function is estimated from fasting or random serum C-peptide levels, or from islet stimulation tests such as a mixed meal tolerance test (MMTT) [17].

Continuous glucose monitoring (CGM) provides an additional tool in the assessment of glycaemic control. The CGM metrics described by the ambulatory glucose profile (AGP) are complementary to HbA_1c_ [18]. Time in range (TIR) has a dose-dependent relationship with higher levels of C-peptide, which are associated with favourable metabolic and clinical outcomes in individuals with type 1 diabetes [7, 19, 20]. There is very limited information on CGM metrics post TPIAT, in particular, in relation to islet secretory function.

Treatment success of islet autotransplantation largely depends on the quantity of transplanted islets and their survival during transplantation and engraftment [6, 21, 22]. However, it is unclear to which degree islet secretory function can be preserved by islet autotransplantation after a total pancreatectomy, and to what extent the degree of islet secretory preservation is related to pre-existing impairments in glucose metabolism. Here we conducted a longitudinal assessment of endocrine function in a Dutch cohort of adult participants undergoing TPIAT and used CGM to assess glycaemic control.

## Methods

This study was reported following the Strengthening the Reporting of Observational Studies in Epidemiology (STROBE) guidelines [23].

### Participant selection

All consecutive individuals that underwent TPIAT from 2014 to 2024 were included in this cohort study. Individuals were referred to our centre from hospitals across the Netherlands. The indication for total pancreatectomy was set by a multidisciplinary team with representatives from the departments of gastroenterology, endocrinology, surgery, radiology and anaesthesiology. In order to be eligible for islet autotransplantation, individuals needed to have a stimulated C-peptide ≥0.6 nmol/l during an MMTT at baseline.

### Total pancreatectomy

All pancreatectomies in this study were total or completion pancreatectomies and are referred to as total pancreatectomies. During the pancreatectomy, the blood vessels were ligated just before pancreas removal to minimise warm ischaemia time. The pancreas was then taken to the back table and flushed with cold Ringer’s Acetate (B. Braun, Melsungen, Germany) supplemented with 7 mmol/l calcium chloride. Subsequently, the pancreas was put in a plastic bag on ice and transported to the in-house good manufacturing practice (GMP) facility for islet isolation. After surgery the participant was taken to the post anaesthesia care unit for observation.

### Islet isolation and transplantation

Islet isolations were performed using an adapted version of the semi-automated method [24, 25]. Digestive enzymes Collagenase NB1 (Serva Electrophoresis, Heidelberg, Germany; Nordmark Biochemicals, Uetersen, Germany) and Neutral Protease (Serve Electrophoresis; Nordmark Biochemicals) were infused intraductally, and when perfusion of the pancreas was insufficient, additional intraparenchymal injections were applied [26]. After complete tissue perfusion, the pancreas was dissected and transferred to the digestion chamber with silicon nitride marbles (Biorep Diabetes, Miami, FL, USA). A Ringer’s Acetate solution supplemented with 7 mmol/l calcium chloride was circulated at 37°C while the chamber was shaken. Digested tissue was collected and washed before storing in Belzer UW cold storage solution (Bridge to Life, Wandsworth, UK) with supplements. A density gradient separation was omitted when the digest volume was considered acceptable for intraportal infusion (up to a maximum of 15 ml). From 2014 to 2025 the islet autotransplantation was preferably performed on the same day as the pancreatectomy. Only intraportal islet transplantation was performed. The portal vein was catheterised by a percutaneous, transhepatic route with x-ray imaging guidance. Participants were heparinised with 70 IU/kg bodyweight. One half of the heparin dose was infused directly into the portal vein and the other half into the transplant bag(s). Portal vein pressure was assessed before, during and after the transplantation. Infusion would begin if the portal vein pressure was <20 mmHg. If despite a 10–20 min pause the portal pressure would remain above 20 mmHg halfway during the infusion, the infusion would be terminated. Upon withdrawal of the catheter, one or more gel foam plugs would be inserted in the hepatic catheter tract to prevent bleeding.

### Peri-transplant care

Intravenous insulin was used post TPIAT with a conversion to subcutaneous insulin when clinically appropriate. During intravenous insulin infusion target glucose concentrations were between 4.0 and 8.0 mmol/l, and during subcutaneous insulin administration between 4.0 and 10.0 mmol/l. A low-carbohydrate diet was implemented for the first 3 months post TPIAT, with a gradual increase in carbohydrate intake from 60 g per day in weeks 1–6, to 80–100 g per day in weeks 7–12, to 100–150 g per day after week 12 in order to reduce the risk of postprandial hyperglycaemic episodes.

### Assessment of islet secretory function and glycaemic control

Islet secretory function was assessed by a 2 h liquid meal stimulation test (MMTT). Participants were required to fast from midnight on the day of the MMTT. If participants used insulin, they were instructed on their insulin regimen several days or weeks before the test in order to reduce the risk of hypo- or hyperglycaemia upon arrival at the LUMC. Target glucose concentrations at the start of MMTT were between 4.0 and 12.0 mmol/l. For participants on basal insulin (either basal insulin only or as part of multiple daily insulin injections), basal insulin was either stopped or continued at the same or an altered dose the day before the MMTT. For participants with an insulin pump, they were asked to put the pump in manual mode with the same fixed dose rate from any time between 00:00 hours and 04:00 hours until the end of the MMTT. Participants consumed a fixed dose of 270 ml of Boost Nutridrink (Danone Nutricia, Zoetermeer, the Netherlands) (*t*=0 min) and blood was drawn for glucose and C-peptide measurement at *t*=−10, 0, 15, 30, 60, 90 and 120 min. C-peptide area under the curve (AUC_C-peptide_) was calculated using the trapezoid method. The ΔC-peptide was defined as the change in concentration of C-peptide from baseline to 30 min.

Since 2019 CGM has been covered by Dutch health insurance for individuals on basal–bolus insulin regimens (injections or pumps). Data were obtained from unblinded CGM devices (Freestyle Libre, Abbot, USA). AGPs of the 14 days prior to the MMTT were collected and included TIR (3.9–10 mmol/l), time in tight range (TITR; 3.9–7.8 mmol/l), time above range (TAR) level 1 (Lv1) (10.1–13.9 mmol/l), TAR level 2 (Lv2) (>13.9 mmol/l), time below range (TBR) Lv1 (3.0–3.8 mmol/l), TBR Lv2 (<3.0 mmol/l), mean glucose (MG), standard deviation (SD) and coefficient of variation (%CV). Based on the AGP, the glucose management indicator (GMI) and glycaemia risk index (GRI) were calculated [27].

We used the BETA-2 score (based on fasting plasma glucose, fasting C-peptide, HbA_1c_, insulin use and body weight) [28] and composite scores derived from Igls criteria [29, 30] as additional measures of clinical graft outcome.

Normal glucose metabolism was defined as HbA_1c_ <39 mmol/mol (5.7%) and a fasting glucose <5.6 mmol/l, prediabetes as HbA_1c_ 39–47 mmol/mol (5.7–6.4%) or a fasting glucose 5.6–6.9 mmol/l, and diabetes mellitus as HbA_1c_ ≥48 mmol/mol (6.5%) or fasting glucose ≥7 mmol/l [31], or requiring glucose-lowering therapy. Severe hypoglycaemic events (SHEs) were defined as hypoglycaemic episodes that required the assistance of a third party.

### Statistical analysis

Data were assessed visually for normality. Comparisons between adjacent timepoints were performed with paired *t* tests. α was set at 0.05. Data are shown in text as mean with SD and in plots as mean with standard error for normally distributed data, as median with IQR for non-normally distributed data and as count with percentage for categorical data. Data on race and ethnicity were not collected. Data on biological sex was collected from the electronic health records. Post hoc, subgroups were created based on pre-TPIAT glycaemic status, and on islet secretory function during the meal stimulation tests. Given the small sample size, we conducted complete-case analyses without imputation. To test the association between CGM metrics and secretion outcomes, linear mixed-effect models with fixed effect for timepoint and random intercept for participants, and quadratic functions for TIR, were used. Analyses were conducted in R version 4.5.1 (2025-06-13 ucrt, http://www.r-project.org) and RStudio version 2025.05.1+513 (Posit Software, PBC, Boston, USA). R packages used are tidyverse, ggplot2, performance, lme4, lmerTest, networkD3, sf and ggpattern.

### Ethics statement

This study was conducted in accordance with the Declaration of Helsinki and used routinely collected healthcare data. All living participants were approached and provided informed consent for use of their data in this study. All data were processed in accordance with applicable privacy legislation (the General Data Protection Regulation [GDPR] and the Dutch GDPR Implementation Act) and analysed in pseudonymised form.

## Results

### Cohort characteristics

We included 26 participants, of whom 19 (73.1%) were female. The mean age was 45.0 ± 13.8 years, and the mean body mass index (BMI) was 24.9 ± 4.5 kg/m^2^. Chronic pancreatitis was the TPIAT indication for 23 (88.5%) participants (Table 1). One participant underwent elective TPIAT because of arterial malformation and two underwent an acute TPIAT: one due to necrotising pancreatitis after iatrogenic perforation of the stomach and the other due to iatrogenic perforation of the duodenum. Participants with chronic pancreatitis had a median disease duration of 5 (IQR 3–9) years before undergoing TPIAT. Twelve participants (46.2%) had undergone previous pancreatic surgery. At baseline, 11 participants (42.3%) had normal glucose metabolism, eight participants (30.8%) had prediabetes (without glucose-lowering therapy) and seven participants (26.9%) had diabetes mellitus. In the subgroup of participants with diabetes mellitus, two were treated with oral glucose-lowering therapy, one with long-acting insulin, one with short-acting insulin and two with combined short- and long-acting insulin, and one was untreated. Participants were transplanted with 4460 ± 2280 islet equivalents (IEQ)/kg, with a median purity of 15% (IQR 8–30) (Table 1, electronic supplementary material [ESM] Table 1). After intraportal transplantation, one participant experienced portal vein thrombosis that was successfully treated with heparin perfusion. No bleeding occurred.

**Table 1:** Participant, islet isolation and transplantation characteristics

Characteristic	Value
Number	26
Female sex	19 (73.1)
Age (years)	45.0 ± 13.8
BMI (kg/m^2^)	24.9 ± 4.5
Indication for TPIAT	
Arterial malformation	1
Iatrogenic gastric/duodenal perforation	2
Chronic pancreatitis	23
Alcohol	8
Genetic	4
Pancreas divisum	2
Biliary	1
Atypical haemolytic uraemic syndrome	1
Idiopathic	7
Duration of pancreatitis (years)	5 (3–9)
Previous pancreas surgery	
None	14 (53.8)
Frey	6 (23.1)
Lateral pancreatectomy	3 (11.5)
Whipple/PPPD	2 (7.7)
Body and tail resection	1 (3.8)
Diabetes mellitus	
No	11 (42.3)
Prediabetes	8 (30.8)
Diabetes	7 (26.9)
Warm ischaemia time (min)	5.0 (1.0–7.4)
Cold ischaemia time (min)	102 (91–126)
Pancreas weight (g)	71.1 ± 29.4
IEQ offered for transplantation	301,087 (187,174–425,543)
IEQ/kg bodyweight	4460 ± 2280
Volume of transplant (ml)	4.85 (3.28–7.50)
Purity of transplant (%)	15 (8–30)
Portal vein pressure (mmHg)	
At start	9.5 ± 4.5
Halfway	11.0 ± 4.3
At end	13.0 ± 4.7

Data are shown as *n* (%), mean ± SD or median (Q1–Q3) PPPD: pylorus-preserving pancreaticoduodenectomy

### Islet graft function

**Fig. 1:**
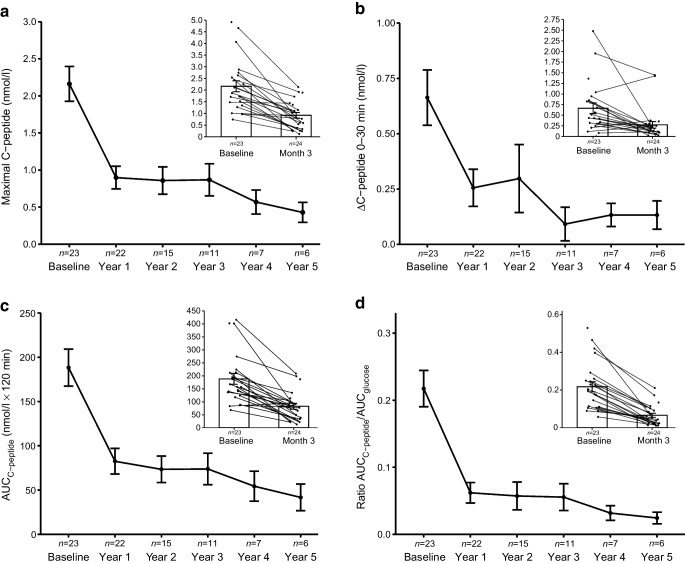
Secretion data obtained during a 2 h MMTT, shown as mean ± standard error. (**a**) Maximal C-peptide. (**b**) ΔC-peptide from baseline to 30 min. (**c**) AUC_C-peptide_ from baseline to 120 min. (**d**) The ratio between AUC_C-peptide_ and AUC_glucose_. Baseline MMTT data were present in only 23 out of 26 participants as three participants underwent TPIAT as an emergency. For each outcome, an accompanying smaller panel illustrates the change from baseline to month 3 for individual participants

At baseline and at 3 months an MMTT was performed in 23 and 24 participants, respectively. Baseline MMTT was missing in three participants because the procedures were performed as an emergency, leaving no opportunity for safe, controlled functional testing. At 3 months, MMTT was missing in two participants because one participant was still hospitalised after an emergency procedure and one participant had died. The AUC_C-peptide_ during a 2 h MMTT was 188.0 ± 99.9 nmol/l at baseline and 82.7 ± 55.8 nmol/l at 3 months (*p*<0.001; Fig. 1). The preservation of insulin secretory capacity in the 22 participants with an MMTT at baseline and 3 months was 45.2 ± 27.0% (ESM Table 2). Maximal stimulated C-peptide decreased from 2.16 ± 1.13 nmol/l at baseline to 0.92 ± 0.59 nmol/l at 3 months (*p*<0.001; islet secretory function preservation 44.0 ± 25.8%) and ΔC-peptide decreased from 0.66 ± 0.60 nmol/l at baseline to 0.27 ± 0.37 nmol/l at 3 months (*p*=0.004; islet secretory function preservation 56.4 ± 59.7%) (ESM Table 2). There was no further statistically significant decline in AUC_C-peptide_, maximal stimulated C-peptide or ΔC-peptide beyond 3 months up to 5 years post TPIAT. At 3 months, retained maximal C-peptide was similar in participants with and without prior pancreatic surgery (45.2 ± 28.6% vs 42.2 ± 22.6%, *p*=0.79), as was retained AUC_C-peptide_ (46.1 ± 30.8% vs 44.0 ± 22.3%, *p*=0.85).

A clinically relevant stimulated C-peptide ≥0.2 nmol/l was reached in 23/24 participants (95.8%) at 3 months, 19/22 participants (86.4%) at 1 year, 14/15 participants (93.3%) at 2 years and 5/6 participants (83.3%) at 5 years post TPIAT.

### CGM parameters and HbA_1c_

**Fig. 2:**
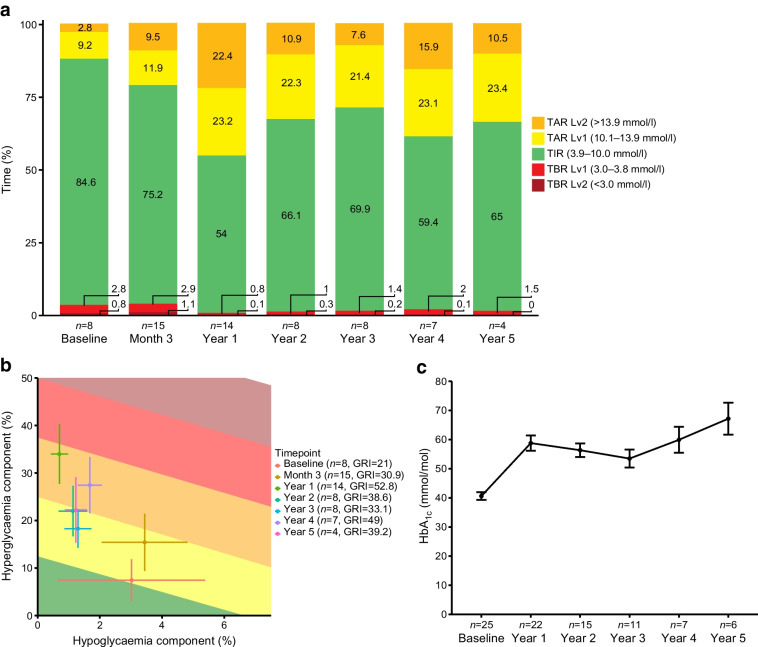
CGM metrics and HbA_1c_ as measures of glycaemic control throughout follow-up. (**a**) AGP describing TIR, TBR Lv1 and Lv2, and TAR Lv1 and Lv2. (**b**) GRI as measure of glycaemic quality, shown as mean ± standard error. Hypoglycaemia component = TBR Lv2 + (0.8 × TBR Lv1); hyperglycaemia component = TAR Lv2 + (0.5 × TAR Lv1); GRI=(3 × hypoglycaemia component) + (1.6 × hyperglycaemia component). (**c**) Serum HbA_1c_ (mmol/mol) levels, shown as mean ± standard error

AGP reports derived from CGM data were available for subsets of participants at baseline and follow-up (Fig. 2). At 3 months the combination of islet graft function, low-carbohydrate dietary intervention, intensive insulin therapy and other instructions for strict glycaemic control resulted in a TIR of 75.2 ± 26.1% (Fig. 2a). There was no increased TBR despite these measures for strict glycaemic control (TBR 3.8 ± 5.7%) (Fig. 2a, ESM Table 3). Furthermore, %CV and GRI were 31.2 ± 9.3% and 30.9 ± 31.0, respectively, at 3 months (Fig. 2b, ESM Table 3). HbA_1c_ increased from 40.6 ± 6.7 mmol/mol to 55.8 ± 24.5 mmol/mol at 3 months (*N*=24, *p*=0.013; Fig. 2c, ESM Table 3).

### Clinical outcome measures

At 3 months, two of 24 (8.3%) participants did not need glucose-lowering therapy, three participants (12.5%) needed only long-acting insulin and 19 participants (79.2%) used a basal–bolus insulin regimen. There was no major change in the use of glucose-lowering therapy beyond 3 months (Table 2). No SHEs were reported throughout follow-up.

**Table 2:** Glucose-lowering therapy

Therapy type	Baseline (*n*=26)	Month 3 (*n*=24)	Year 1 (*n*=22)	Year 2 (*n*=15)	Year 3 (*n*=12)	Year 4 (*n*=7)	Year 5 (*n*=6)
No medication	20 (76.9)	2 (8.3)	2 (9.1)	1 (6.7)	1 (8.3)	0 (0)	0 (0)
Only non-insulin glucose-lowering therapy	2 (7.7)	0 (0)	2 (9.1)	1 (6.7)	0 (0)	0 (0)	0 (0)
Bolus insulin^a^	1 (3.8)	1 (4.2)	4 (18.2)	4 (26.7)	6 (50.0)	4 (57.1)	4 (66.7)
Basal insulin^a^	2 (7.7)	3 (12.5)	3 (13.6)	2 (13.3)	1 (8.3)	1 (14.3)	1 (16.7)
Basal–bolus insulin^a^	1 (3.8)	18 (75.0)	11 (50.0)	7 (46.7)	4 (33.3)	2 (28.6)	1 (16.7)
Insulin/kg bodyweight, IU/kg	0.12 ± 0.08	0.39 ± 0.48	0.35 ± 0.24	0.26 ± 0.09	0.25 ± 0.13	0.25 ± 0.11	0.37 ± 0.35

Data are shown as *n* (%) or mean ± SD

^a^With or without non-insulin glucose-lowering therapy

**Fig. 3:**
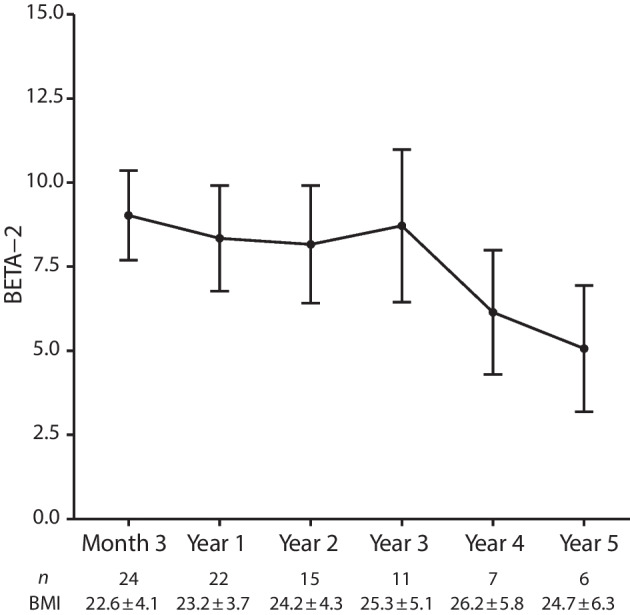
BETA-2 score calculated from fasting C-peptide, fasting glucose, HbA_1c_ and insulin dose corrected for body weight. Number of participants and BMI (mean ± SD) are shown

The BETA-2 score was 9.0 ± 6.5 at 3 months (Fig. 3). Beyond 3 months there were no significant changes in BETA-2 score (range *p*=0.07 to *p*=0.91). Adapted novel composite clinical outcome scores based on the Igls criteria that could also be applied to islet autotransplantation recipients have been proposed [29, 30] and are presented in ESM Table 4 and ESM Fig. 1.

### Effect of pre-TPIAT glycaemic status on graft function and clinical outcome and effect of post-TPIAT graft function on glycaemic control and clinical outcome

In post hoc subgroup analyses at 3 months, participants were divided by pre-TPIAT glycaemic status. AUC_C-peptide_ preservation was 52.9 ± 33.4% in participants with normal glucose metabolism, 43.9 ± 21.3% in participants with prediabetes and 39.1 ± 28.3% in participants with diabetes (*p*=0.65). For maximal C-peptide this was 49.2 ± 25.9%, 46.0 ± 27.2% and 36.5 ± 25.9%, respectively (*p*=0.65). ESM Fig. 2 shows the relationship between baseline metabolic status, IEQ transplanted and AUC_C-peptide_.

**Fig. 4:**
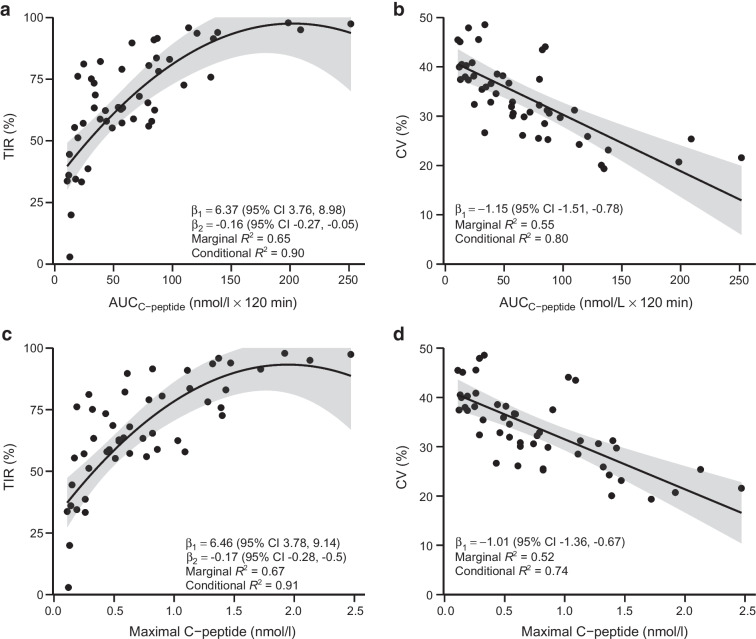
Association between MMTT-derived C-peptide secretion and CGM metrics during follow-up. Pooled observations from month 3 to year 5 (baseline excluded) are shown. (**a**) AUC_C-peptide_ (nmol/l × 0–120 min) vs TIR (%). β_1_ and β_2_ represent the linear and quadratic associations, between AUC_C-peptide_ and TIR, per 10 units increase of AUC_C-peptide_. (**b**) AUC_C-peptide_ vs glucose %CV. β_1_ represents the change in %CV for each 10 units of AUC_C-peptide_. (**c**) Maximal C-peptide (nmol/l) vs TIR. β_1_ and β_2_ represent the linear and quadratic associations, respectively, between maximal C-peptide and TIR, per 0.1 units increase in maximal C-peptide. (**d**) Maximal C-peptide vs %CV. β_1_ represents the change in %CV for each 0.1 units of maximal C-peptide. Curves represent fixed-effects predictions from random-intercept linear mixed-effects models including timepoint as a fixed effect; shaded areas denote 95% CIs

At 3 months participants with the highest AUC_C-peptide_ tertile pre-TPIAT had 95.1 ± 2.7% TIR and 22.4 ± 2.9 %CV while participants in the lowest AUC_C-peptide_ tertile pre-TPIAT had 53.6 ± 33.4% TIR and 40.1 ± 6.7 %CV (*p*=0.05 and *p*=0.002, respectively). Beyond 3 months the effect of better graft function on glycaemic parameters persisted. ESM Fig. 3 shows the change in function over time grouped per tertile of AUC_C-peptide_, maximal C-peptide, ΔC-peptide and ratio of AUC_C-peptide_ to AUC_glucose_ pre-TPIAT. At all time points post TPIAT, TIR correlated positively with AUC_C-peptide_ and maximal C-peptide, whereas %CV correlated negatively with AUC_C-peptide_ and maximal C-peptide. Adjusted for timepoint and accounting for repeated measures, each 10 U increase in AUC_C-peptide_ was associated with an average 6.4 %pt increase in TIR (95% CI 3.8, 9.0; *p*<0.0001; with quadratic β −0.16; 95% CI −0.05, −0.27) and a 1.2 %pt decrease in %CV (95% CI 0.8, 1.5; *p*<0.0001). Similarly, for each 0.1 nmol/l increase in maximal C-peptide, TIR increased by 6.4 %pt (95% CI 3.8, 9.1; *p*<0.0001; with quadratic β −0.17; 95% CI −0.05, 0.28) and %CV decreased by 1.0 %pt (95% CI 0.7, 1.4; *p*<0.0001; Fig. 4).

At 3 months, basal–bolus insulin regimens were used in 100% of participants with pre-TPIAT prediabetes, in 85.7% of participants with diabetes and in 44.4% of participants with normal glucose metabolism.

## Discussion

Here we show that after TPIAT there is approximately 45% preservation of islet secretory function, as measured by the AUC_C-peptide_ during a liquid mixed meal stimulation test. These results can help in shared decision making to discuss the expected islet graft function and associated clinical benefits. Importantly, our study provides direct clinical quantification of functional islet preservation, extending earlier PET-based observations [32] and preclinical reports [33–35] that an estimated 50–70% of islets are lost during isolation, transplantation and engraftment.

Historically, TPIAT was mainly performed in individuals without diabetes or previous pancreatic surgery in order to maximise the chance for insulin independence as the preferred outcome [5, 36–38]. However, clinical benefit is already observed at low secretory capacity in type 1 diabetes [8] and after islet transplantation [39]. A C-peptide as low as 0.04–0.07 nmol/l is associated with protection against severe hypoglycaemia and microvascular complications [7, 8, 39, 40]. We accepted individuals for TPIAT in the LUMC with a stimulated C-peptide equal to or greater than 0.6 nmol/l during a liquid meal stimulation test. Our cohort included a high percentage of individuals with previous pancreatic surgery (46.2%) and prediabetes or diabetes mellitus (57.7%). Both factors are known to reduce the quantity and quality of islets available for transplantation [41, 42]. Nevertheless, in our study nearly all participants still achieved a clinically relevant stimulated C-peptide concentration of ≥0.2 nmol/l [7]. What concentration of stimulated C-peptide before pancreatectomy and anticipated benefit are required for justifying the risks and costs of the islet autotransplantation procedure is still an open question. Other aspects such as BMI and degree of insulin requirement may also play a role in this decision. But based on the data showing that even low concentrations of C-peptide are associated with clinical benefit, in particular prevention of SHEs, all individuals that undergo total pancreatectomy due to non-malignant pancreatic disease should at least be considered for TPIAT. Our findings demonstrate that TPIAT can provide clinically meaningful preservation of islet function even in individuals with significant pancreatic disease or previous surgery, supporting a wider application of the procedure given its low procedure-related risk.

Participants with prediabetes and diabetes mellitus before TPIAT had no significantly different preservation of islet secretory function compared with participants with normal glucose metabolism, although numbers were small, making robust conclusions difficult.

The high prevalence rates of prediabetes, diabetes mellitus and previous pancreatic surgery before TPIAT are likely to explain the need for insulin in the majority of our group of individuals, necessitating routine follow-up for glycaemic control and diabetes-related complications. Post-TPIAT insulin requirements were higher in individuals with existing diabetes mellitus. This may be in part due to a lower transplanted islet dose, lower islet quality and higher insulin resistance [43].

After the first 3 months post TPIAT, C-peptide secretion did not significantly change, also reflected by composite clinical outcome scores based on Igls criteria [29, 30], which is in line with previous reports [15, 44, 45]. Moreover, in our cohort the relative change in AUC_C-peptide_ observed after 3 months was similar to that reported in the Minnesota cohort [15]. Although the MMTT protocol was standardised within our cohort, comparisons with other cohorts may be affected by differences in drink composition and dosing of the liquid meal.

As this study is among the first to assess longitudinal glycaemic control in a TPIAT cohort using CGM, comparisons with other cohorts are limited and challenging [46, 47]. The recommended TIR target ≥70% was not consistently achieved. This may in part reflect low diabetes-related health literacy shortly after TPIAT [48, 49]. However, mean TBR was maintained at the recommended <4%. In addition, no SHEs were observed, indicating that individuals undergoing TPIAT in our cohort were protected from clinically significant hypoglycaemia. However, our study did not include a control group of individuals with TP. As major technological advances (glucose sensors, insulin pumps, algorithms for automated insulin delivery) have been made that reduce the risk of severe hypoglycaemia [50], studies comparing the effects of total pancreatectomy-only with TPIAT on the incidence of SHEs and other relevant clinical measures are needed. Treatment goals for glycaemic variability (%CV <36%) [47] were attained up until the third year in the majority of participants, and mean %CV was seemingly lower than in a sample of individuals from a post-TP cohort [43], further strengthening that TPIAT offers glycaemic benefit over TP alone [3].

Strengths of this study are the inclusion of all individuals in the Netherlands that have undergone TPIAT in the last 10 years, the considerable number of participants with preoperative prediabetes or diabetes and previous pancreatic surgery, the protocolised liquid meal stimulation tests at baseline and during the postoperative follow-up allowing detailed insight into preservation of islet secretory function and the use of CGM metrics that provide more detailed insight into glycaemic control post TPIAT. An important limitation of the study is the limited number of participants. In combination with the imbalance in follow-up between participant groups based on tertiles in secretory function before TPIAT, this has likely influenced clinical outcome results beyond 3 years, with autoimmunity not being an adverse factor in the autologous transplantation setting. This impairs our ability to draw firm conclusions on differences between subgroups and on long-term outcomes.

In conclusion, individuals undergoing TPIAT, including those with prediabetes or diabetes mellitus, show consistent and clinically meaningful preservation of islet secretory function across different measures of stimulated C-peptide.

## Supplementary Information

Below is the link to the electronic supplementary material.

ESM (PDF 363 KB)

## Data Availability

The data that support the findings of this study are not openly available due to reasons of sensitivity and may be shared by the corresponding author upon reasonable request. Data are located in controlled access data storage at Leiden University Medical Center.

## Abbreviations

AGP: Ambulatory glucose profile
AUC_C-peptide_: C-peptide area under the curve
BMI: Body mass index
CGM: Continuous glucose monitoring
%CV: Coefficient of variation
GDPR: General Data Protection Regulation
GRI: Glycaemia risk index
IEQ: Islet equivalents
Lv1: Level 1
Lv2: Level 2
MMTT: Mixed meal tolerance test
SD: Standard deviation
SHE: Severe hypoglycaemic event
TAR: Time above range
TBR: Time below range
TIR: Time in range
TPIAT: Total pancreatectomy with islet autotransplantation
