# Supplementary material for: Degree of islet function preservation and continuous glucose monitoring in individuals undergoing total pancreatectomy with islet autotransplantation
Source: Diabetologia. 2026 Jun 27;69(9):2487–97. doi: 10.1007/s00125-026-06772-9 (PMC13424119; doi:10.1007/s00125-026-06772-9)
Supplement: Supplementary file 1 — ESM (PDF 363 KB) [file 125_2026_6772_MOESM1_ESM.pdf]

Electronic Supplementary Material (ESM)

Degree of islet function preservation and continuous glucose monitoring in individuals undergoing total pancreatectomy with islet autotransplantation

Maarten C. Tol[1,2], Willemijn E. M. E. de Vos[1,2], Ian P. J. Alwayn[2,3], J. Sven D. Mieog[3], Jeanin E. van Hooft[2,4], Akin Inderson[2,4], Marieke Niesters[5], Marieke D. Hellinga[5], Michiel F. Nijhoff[1,2], Arian R. van Erkel[6], Marten A. Engelse[1,2], Volkert A. L. Huurman[2,3], Eelco J. P. de Koning[1,2]

[1]Department of Internal Medicine, Leiden University Medical Center, Leiden, the Netherlands

[2]LUMC Transplant Center, Leiden University Medical Center, Leiden, the Netherlands

[3]Department of Surgery, Leiden University Medical Center, Leiden, the Netherlands

[4]Department of Gastroenterology and Hepatology, Leiden University Medical Center, Leiden, the Netherlands

[5]Department of Anaesthesiology, Leiden University Medical Center, Leiden, the Netherlands

[6]Department of Radiology, Leiden University Medical Center, Leiden, the Netherlands

Corresponding author:

Eelco J. P. de Koning,

Email: e.j.p.de_koning@lumc.nl

ESM Table 1 Individual islet equivalents (IEQ) transplanted. Each row represents a unique patient.

Patient	IEQ transplanted	IEQ transplanted per kg	Insulin independence at 1 year
1	140,040	2,406	No
2	306,522	3,035	Yes
3	73,043	1,461	No
4	106,043	1,414	No
5	208,696	3,478	No
6	239,130	3,223	No
7	393,478	5,317	Yes
8	213,043	3,088	No
9	150,815	2,320	No
10	235,271	3,410	No
11	295,652	3,963	No
12	717,391	8,912	N/A
13	721,739	8,966	Yes
14	422,283	4,968	No
15	407,609	4,648	No
16	326,050	5,771	No
17	728,261	7,508	No
18	426,630	5,491	N/A
19	157,609	2,866	No
20	476,087	6,801	Yes
21	391,304	4,715	No
22	213,043	3,464	N/A
23	141,404	2,209	No
24	852,714	8,966	No
25	163,043	1,976	No
26	570,652	5,672	N/A

IEQ: islet equivalents. N/A: not applicable.

ESM Table 2 Characterization of islet secretory function during a mixed meal tolerance test before and after TPIAT

	Baseline	Month 3	Year 1	Year 2	Year 3	Year 4	Year 5
**Maximal stimulated C-peptide (nmol/l)**	2.16 ± 1.13	0.92 ± 0.59	0.9 ± 0.72	0.86 ± 0.71	0.87 ± 0.72	0.57 ± 0.43	0.43 ± 0.33
	(n=23)	(*n*=24)	(*n*=22)	(*n*=15)	(*n*=11)	(*n*=7)	(*n*=6)
preserved function compared to baseline (%)	*Ref*	44.0 ± 25.8	41.6 ± 27.9	44.5 ± 31.5	51.8 ± 31.4	36.7 ± 28.5	33.6 ± 15.1
		(*n*=22)	(*n*=20)	(*n*=13)	(*n*=9)	(*n*=6)	(n=6)
preserved function compared to 3 months (%)	*N/A*	*Ref*	86.0 ± 30.7	89.6 ± 34.2	93.7 ± 40.6	66.3 ± 29.4	77.7 ± 38.4
			(*n*=21)	(*n*=14)	(*n*=10)	(*n*=7)	(*n*=6)
**ΔC-peptide 0-30 min (nmol/l)**	0.66 ± 0.6	0.27 ± 0.37	0.26 ± 0.39	0.3 ± 0.6	0.09 ± 0.25	0.13 ± 0.14	0.13 ± 0.16
	(*n*=23)	(*n*=24)	(*n*=22)	(*n*=15)	(*n*=11)	(*n*=7)	(*n*=6)
preserved function compared to baseline (%)	*Ref*	56.4 ± 59.7	59.5 ± 88.4	70.6 ± 103.8	45.2 ± 76.0	70.4 ± 88.9	59.2 ± 79.6
		(*n*=21)	(*n*=20)	(*n*=13)	(*n*=9)	(*n*=6)	(*n*=6)
preserved function compared to 3 months (%)	*N/A*	*Ref*	80.0 ± 42.5	104.8 ± 81.6	73.5 ± 52.1	72.5 ± 66.5	96.9 ± 78.0
			(*n*=21)	(*n*=14)	(*n*=10)	(*n*=7)	(*n*=6)
**$AUC_{C\text{-peptide}}$ (nmol/l /times × 120 min)**	188.3 ± 99.9	82.7 ± 55.8	82.5 ± 67.9	73.4 ± 57.7	73.8 ± 59.1	54.3 ± 44.7	41.7 ± 36.9
	(*n*=23)	(*n*=24)	(*n*=22)	(*n*=15)	(*n*=11)	(*n*=7)	(*n*=6)
Preserved function compared to baseline (%)	*Ref*	45.2 ± 27.0	43.3 ± 27.9	42.2 ± 26.7	47.4 ± 28.1	39.5 ± 29.9	35.9 ± 19.5
		(n=22)	(*n*=20)	(*n*=13)	(*n*=9)	(*n*=6)	(*n*=6)
preserved function compared to 3 months (%)	*N/A*	*Ref*	87.9 ± 35.1	87.7 ± 36.5	89.5 ± 49.4	67.1 ± 36.6	84.9 ± 57.3
			(*n*=21)	(*n*=14)	(*n*=10)	(*n*=7)	(*n*=6)

Ref: reference value. N/A: Not applicable.

ESM Table 3 Glycaemic outcomes measures derived from continuous glucose monitoring and HbA_{1c}.

	Baseline (*n*=8)	Month 3 (*n*=15)	Year 1 (*n*=14)	Year 2 (*n*=8)	Year 3 (*n*=8)	Year 4 (*n*=7)	Year 5 (*n*=4)
TIR (3.9-10 mmol/l), %	84.6 ± 18.3	75.2 ± 26.1	54.0 ± 23.7	66.1 ± 17.5	69.9 ± 15.3	59.4 ± 17.7	65.0 ± 17.7
TITR (3.9-7.8 mmol/l), %	66.9 ± 26.9	55.2 ± 24.4	31.2 ± 19.1	39.1 ± 16.6	45.3 ± 17.9	36.4 ± 16.9	40.6 ± 17.3
TAR (> 10.0 mmol/l), %	12.1 ± 19.4	21.3 ± 25.1	45.6 ± 23.7	33.1 ± 17.4	29.0 ± 16.4	39.0 ± 17.5	33.9 ± 17.9
TAR Lv1 (10.1-13.9 mmol/l), %	9.2 ± 14.1	11.9 ± 10.1	23.2 ± 8.1	22.3 ± 8.3	21.4 ± 10.6	23.1 ± 6.0	23.4 ± 9.1
TAR Lv2 (> 13.9 mmol/l), %	2.8 ± 5.6	9.5 ± 22.5	22.4 ± 24.2	10.9 ± 13.4	7.6 ± 7.1	15.9 ± 14.1	10.5 ± 10.0
TBR (<3.9 mmol/l), %	3.5 ± 7.5	3.8 ± 5.7	0.8 ± 1.2	1.3 ± 1.4	1.6 ± 1.5	2.0 ± 1.2	1.5 ± 0.9
TBR Lv1 (3.0-3.8 mmol/l), %	2.8 ± 5.7	2.9 ± 4.0	0.8 ± 1.2	1.0 ± 1.0	1.4 ± 1.4	2.0 ± 1.2	1.5 ± 0.9
TBR Lv2 (< 3.0 mmol/l), %	0.8 ± 2.1	1.1 ± 2.6	0.1 ± 0.1	0.3 ± 0.5	0.2 ± 0.2	0.1 ± 0.1	0.0 ± 0.1
Mean glucose, mmol/l	7.1 ± 1.9	8.4 ± 3.4	10.9 ± 3.3	9.2 ± 1.7	8.6 ± 1.4	9.7 ± 1.9	9.2 ± 1.6
SD glucose, mmol/l	1.8 ± 0.8	2.7 ± 1.7	3.7 ± 1.5	3.1 ± 1.0	2.9 ± 0.8	3.7 ± 1.2	3.4 ± 1.4
CV, %	24.4 ± 5.7	31.2 ± 9.3	33.8 ± 7.9	32.9 ± 5.5	33.5 ± 4.6	37.1 ± 5.7	36.2 ± 9.1
CV < 36%, *n* (%)	8 (100)	10 (66.7)	8 (57.1)	6 (75)	5 (62.5)	2 (28.6)	2 (50)
GRI	21.0 ± 23.6	30.9 ± 31.0	52.8 ± 30.5	38.6 ± 24.5	33.1 ± 16.3	49.0 ± 25.3	39.2 ± 22.2
GMI, %	6.3 ± 0.8	6.9 ± 1.5	8.0 ± 1.4	7.3 ± 0.7	7.0 ± 0.6	7.5 ± 0.8	7.3 ± 0.7
Time sensor active, %	67.8 ± 36.0	79.9 ± 21.7	89.1 ± 10.3	93.3 ± 6.9	92.8 ± 5.5	82.7 ± 30.6	97.7 ± 1.0
HbA_{1c}, mmol/mol (*n*)	40.6 ± 6.7 (25)	55.8 ± 24.5 (24)	58.8 ± 12.4 (22)	56.4 ± 9.0 (15)	53.5 ± 10.1 (11)	59.9 ± 11.8 (7)	67.2 ± 13.4 (6)

Data in mean ± SD unless otherwise indicated. TIR: time in range; TITR: time in tight range; TAR: time above range; TBR: time below range; SD: standard deviation; %CV: coefficient of variation; GRI: glycaemia risk index; GMI: glucose management indicator.

ESM Table 4 Novel composite clinical outcome scores.

	Baseline (*n*=23)		Month 3 (*n*=24)		Year 1 (*n*=22)		Year 2 (*n*=15)		Year 3 (*n*=11)		Year 4 (*n*=7)		Year 5 (*n*=6)	
	Auto-Igls	Igls 2.0	Auto-Igls	Igls 2.0	Auto-Igls	Igls 2.0	Auto-Igls	Igls 2.0	Auto-Igls	Igls 2.0	Auto-Igls	Igls 2.0	Auto-Igls	Igls 2.0
**Optimal**	18 (78.3%)	17 (73.9%)	1 (4.2%)	2 (8.3%)	1 (4.5%)	4 (18.2%)	2 (13.3%)	2 (13.3%)	1 (9.1%)	1 (9.1%)	0	0	0	0
**Good**	3 (13%)	6 (26.1%)	17 (70.8%)	22 (91.7%)	6 (27.3%)	16 (72.7%)	2 (13.3%)	13 (86.7%)	5 (45.5%)	10 (90.1%)	2 (28.6%)	6 (85.7%)	1 (16.7%)	5 (83.3%)
**Marginal**	2 (8.7%)	0	5 (20.8%)	0	12 (54.5%)	2 (9.9%)	11 (73.3%)	0	5 (45.5%)	0	4 (57.1%)	1 (14.3%)	4 (66.7%)	1 (16.7%)
**Failed**	0	0	1 (4.2%)	0	3 (13.6%)	0	0	0	0	0	1 (14.3%)	0	1 (16.7%)	0

Two novel composite clinical outcome scores that can be used in autologous islet transplantation. Auto-Igls score, as proposed by McEachron et al.[30], that is scored using HbA_{1c}, severe hypoglycaemic events, insulin dose and C-peptide (stimulated or fasting), and the Igls criteria 2.0 for beta cell graft function, as proposed by Landstra et al.[29] using the concentration of C-peptide (fasting or stimulated) and the use of insulin or noninsulin glucose-lowering therapy.

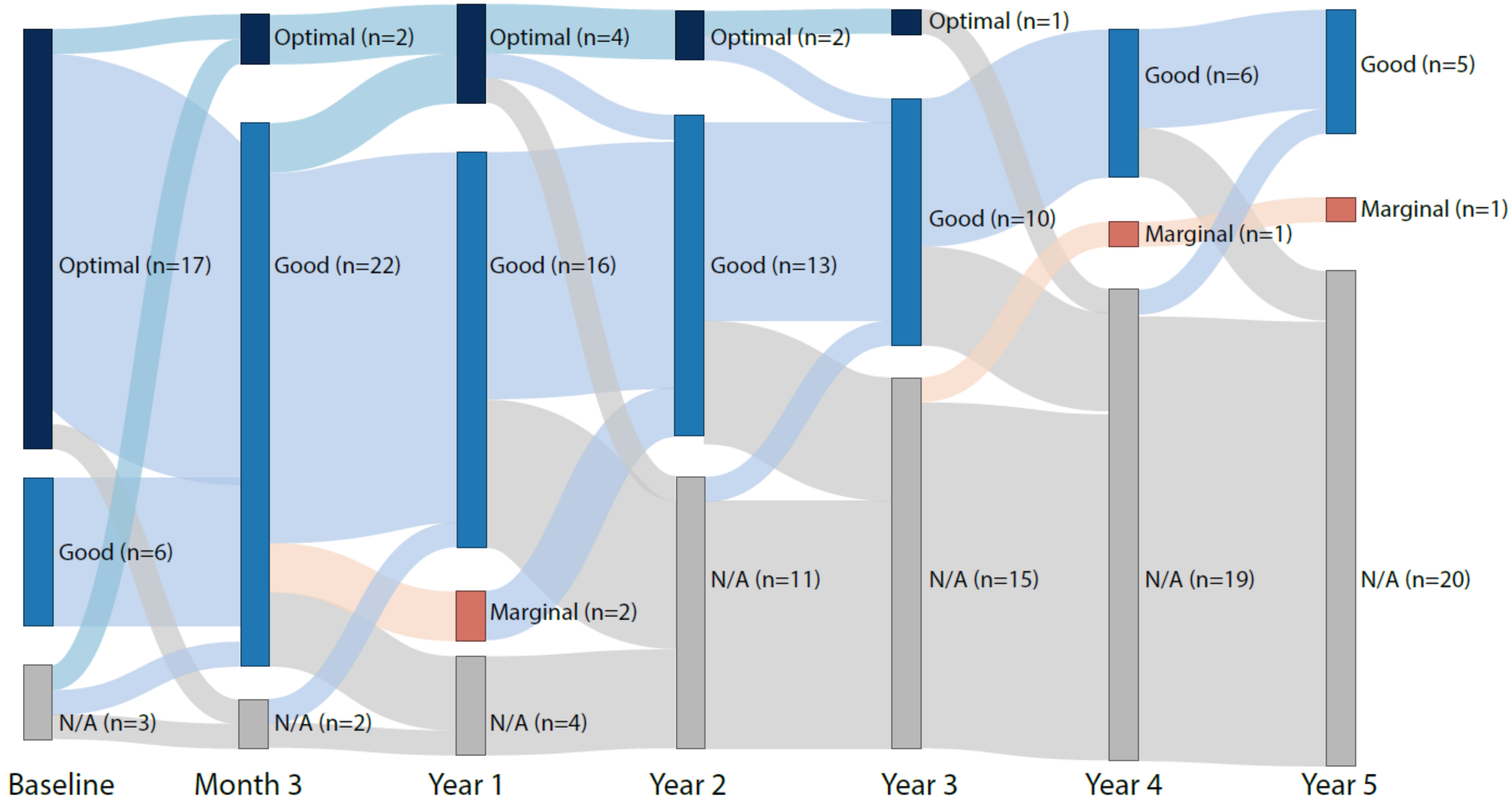

ESM Fig. 1 Igls criteria 2.0 for beta-cell graft function, as proposed by Landstra et al. [29], scored using the concentration of C-peptide (fasting or stimulated) and the use of insulin or noninsulin glucose-lowering therapy. Flows summarize transitions of patients between Igls criteria 2.0 beta-cell graft function categories over time.

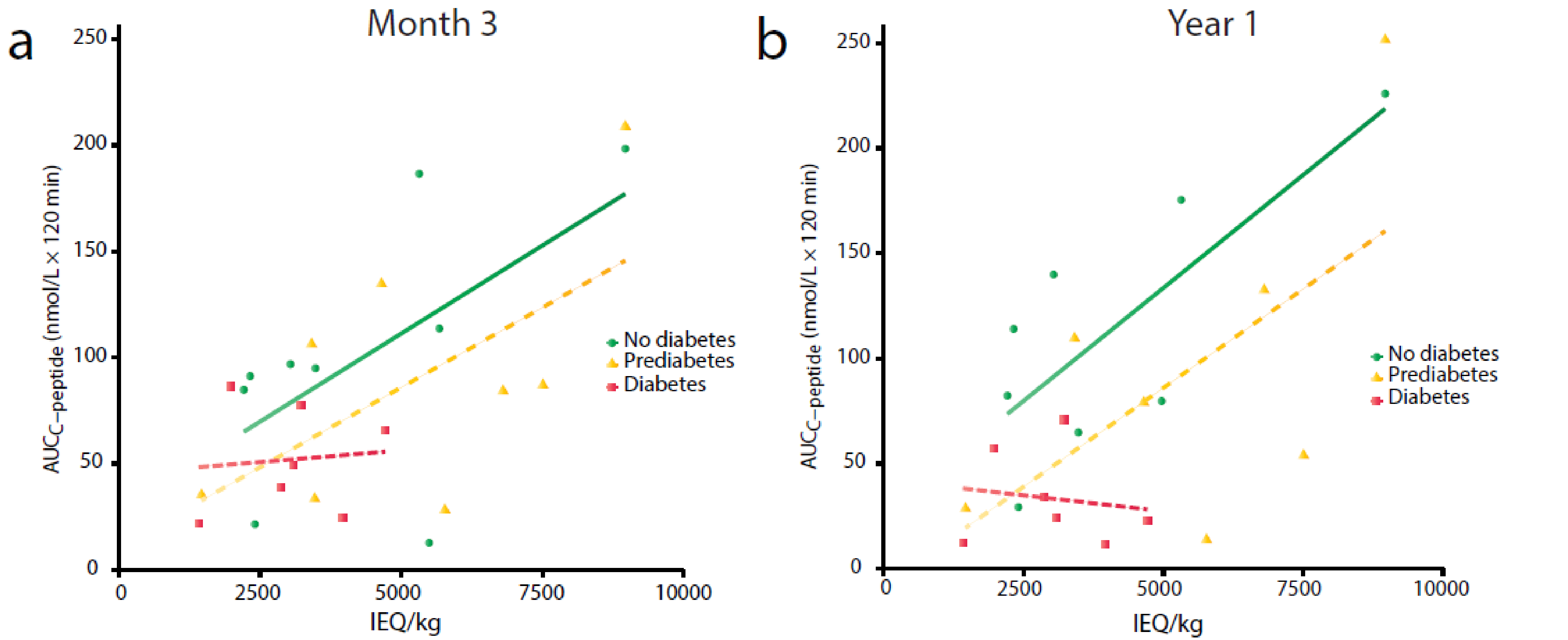

ESM Fig. 2 The relationship between transplanted islet equivalents transplanted per kilogram bodyweight and (A) $AUC_{C\text{-peptide}}$ at 3 months and (B) $AUC_{C\text{-peptide}}$ at 1 year. No diabetes: green circles and solid line; prediabetes: orange triangles and wide dashed line; diabetes: red squares with narrow dashed line.

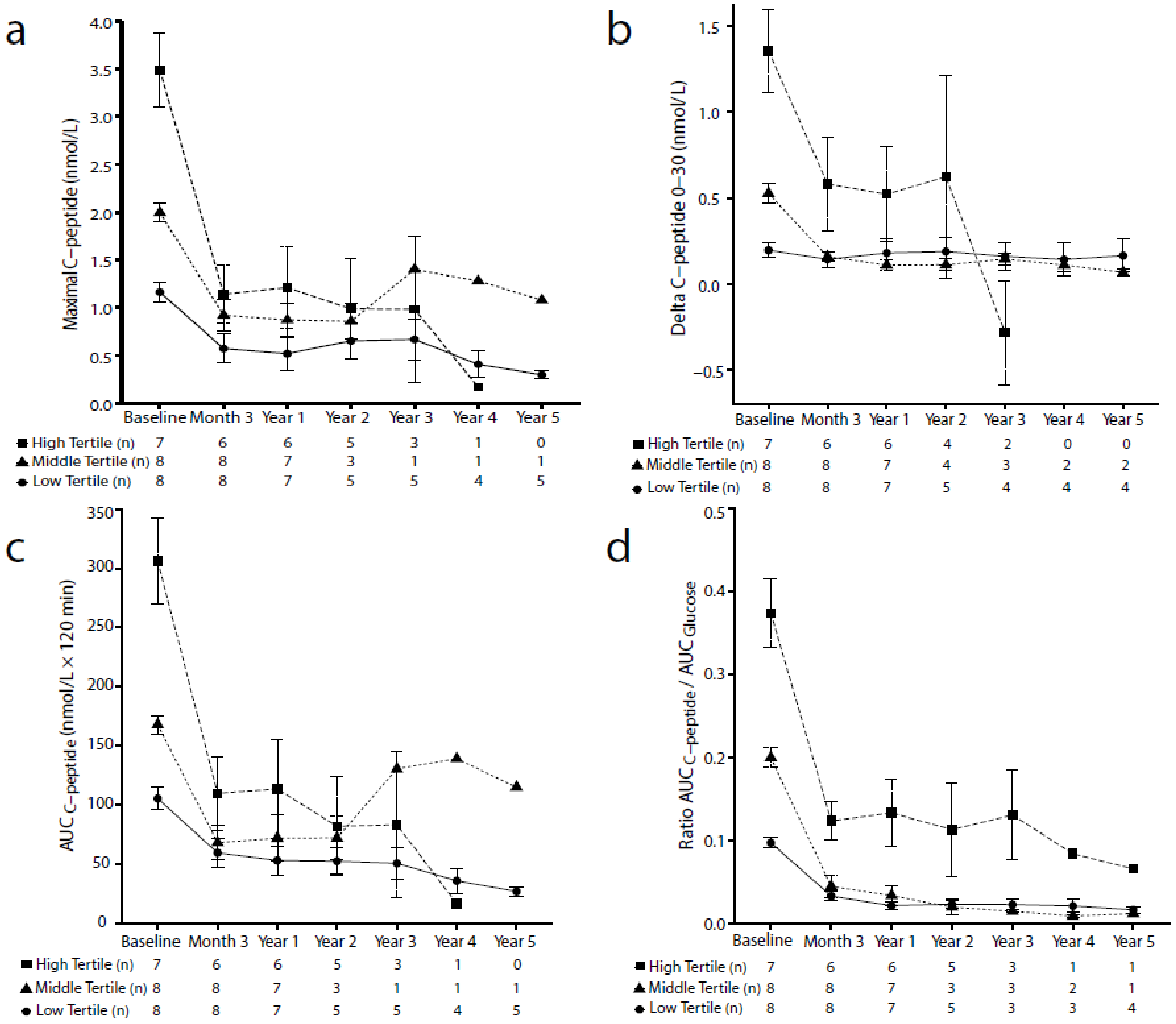

ESM Fig. 3 *Secretion data obtained during a two-hour Mixed Meal Tolerance Test (MMTT), shown as mean ± standard error. Patients were stratified into low, middle and high tertiles based on the baseline value of the respective functional measure. A) Maximal C-peptide. B) ΔC-peptide from baseline to 30 minutes. C) AUC$_{C\text{-peptide}}$ from baseline to 120 minutes. D) The ratio between AUC$_{C\text{-peptide}}$ and AUC$_{Glucose}$.*
